# *AVPR1A* RS3 and relationship maintenance processes in newlywed couples

**DOI:** 10.3389/fpsyg.2025.1303065

**Published:** 2025-03-03

**Authors:** Anastasia Makhanova, James K. McNulty, Lisa A. Eckel, Larissa Nikonova, Jennifer A. Bartz, Arial S. Bloshinsky, Elizabeth A. D. Hammock

**Affiliations:** ^1^Department of Psychological Science, University of Arkansas, Fayetteville, AR, United States; ^2^Department of Psychology, Florida State University, Tallahassee, FL, United States; ^3^Department of Psychology, McGill University, Montreal, QC, Canada

**Keywords:** vasopressin receptor gene, RS3 polymorphism, pair bonding, marital problems, marriage

## Abstract

**Introduction:**

Maintaining relationship quality during the first few years can be difficult for many couples. We examined whether variability in the repeat-length polymorphism RS3 on the vasopressin receptor gene *AVPR1A* is associated with relationship maintenance processes and trajectories of marital satisfaction over the first three years of marriage.

**Methods:**

Newlywed couples (*N*=70; 128 individuals) reported on various aspects of their marriage within three months of their wedding and on their marital satisfaction every four months for three years, and provided saliva samples that we genotyped for RS3 alleles. Based on the literature, we predicted that people with at least one copy of target allele 334 (vs. none) would report more problems in pair bonding. We also used another genotype analysis approach from the extant literature, by testing whether people with a greater (vs. fewer) number of short alleles would report more problems in pair bonding.

**Results:**

Across both approaches, results failed to support our predictions. In fact, the significant effects that did emerge were in the opposite direction from our predictions: people with at least one copy of allele 334 reported *fewer* marital problems and less interest in romantic alternatives; the number of short alleles was similarly *positively* associated with more dedication to the relationship and greater relationship satisfaction at the beginning of marriage.

**Discussion:**

Discrepancies between these findings and prior research illustrate the challenges of candidate gene studies with small sample sizes. Nevertheless, in offering a potential reconciliation between the discrepancies, we suggest that attending to relational phase may be critical to understanding the role of RS3 in couple functioning; *AVPR1A* RS3 variability may be differentially associated with pair bonding in the newlywed stage compared to established marriages.

## 1 Introduction

On their wedding day, couples say “I do” with the intention and promise of being together forever. However, 30 to 50% of couples divorce (Amato and James, 2010), and even those who stay together experience fluctuations and even declines in marital satisfaction over time (Joiner et al., 2024). Considering the robust link between marital satisfaction and health (Kiecolt-Glaser, 2018; Robles et al., 2014; Sbarra et al., 2011), understanding the specific factors that can undermine the strength of a pair bond is paramount.

Theoretical perspectives and extant research indicate that individual differences contribute to cognitions and behaviors that can predict the maintenance or deterioration of marital satisfaction (Karney and Bradbury, 1995; McNulty et al., 2021). In addition to well-established psychological factors (e.g., neuroticism), research suggests that biological factors may also play a key role in maintaining pair bonds. In particular, as detailed below, research in non-human animals and humans indicates that variation on the repeat polymorphism RS3 on the arginine vasopressin receptor 1a (*AVPR1A*) gene may be one important genetic source of individual differences in relationship maintenance process because it has been linked to sociosexual processes in both nonhumans and humans. To test this question, we leveraged data from a longitudinal study of newlywed couples to examine whether variability on RS3 is associated with relationship maintenance processes and the trajectory of marital satisfaction over the first three years of marriage.

### 1.1 *AVPR1A* and sociosexual processes

Research using monogamous prairie voles has offered important insights for the mechanisms of pair bond formation and maintenance. Unlike other vole species (e.g., meadow voles), prairie voles form long-term pair bonds and rear offspring together. Studies using a variety of techniques have provided strong evidence that the vasopressin system, including the vasopressin 1a receptors, plays an important role in preference for a long-term partner (Young and Wang, 2004). Specifically, this research indicates that prairie voles differ from meadow voles in terms of the brain’s distribution of vasopressin’s primary receptor, AVPR1A (Insel and Hulihan, 1995). Moreover, other research indicates that experimentally increasing AVPR1A density in specific brain areas in meadow voles facilitates pair bonding (Lim et al., 2004).

One difference in the vasopressin receptor genes between meadow and prairie voles that may explain the differential distribution of vasopressin receptors in the brain is a repetitive expansion at a particular microsatellite that is considerably longer in prairie voles than in meadow voles (Young et al., 1999). Indeed, this repetitive microsatellite has been functionally linked to regulation of gene expression in voles (Hammock and Young, 2004) and a similar pattern of repetitive microsatellite on *AVPR1A* has been found in hominids. Humans and bonobos—species with sociosexual bonding—are more similar to each other at this locus than either is to the common chimpanzee—which lacks sociosexual bonding (Savage-Rumbaugh and Wilkerson, 1978). Unlike humans and bonobos, some chimpanzees have a single microsatellite, whereas others have a duplicated microsatellite (Donaldson et al., 2008; Hammock and Young, 2005; Staes et al., 2014). Critically, such within-species allelic variation has been implicated in within species variation in social behavior (Hopkins et al., 2012; Hopkins et al., 2014) and brain anatomy (Mulholland et al., 2020). Consequently, this repeat region has generated interest in understanding genetic contributions to individual differences in species-typical social behavior, including sociosexual bonding behavior in species demonstrating this phenotype.

In humans, the *AVPR1A* gene has both single nucleotide and repeat polymorphisms, and research has begun examining whether variation in the length of four identified repeat regions—GT_25_, RS3, RS1, AVR (Landefeld et al., 2018)—is associated with human social behavior (Kim et al., 2002; Wassink et al., 2004; Yirmiya et al., 2006). The most consistent associations emerge for the polymorphism RS3, which is a complex (CT)_4_-TT-(CT)_8_-(GT)_24_ repeat. At single nucleotide polymorphisms (SNPs), individuals differ by a single nucleotide substitution; at repeat polymorphisms, in contrast, individuals differ in the length of the sequence of nucleotides in this region as well as possible nucleotide substitutions. Depending on the primers used to amplify this region, repeat lengths on RS3 can range from 308 to 343 base pairs (Knafo et al., 2008) or 318 to 352 base pairs (Kim et al., 2002; Meyer-Lindenberg et al., 2009; Walum et al., 2008). That is, people have two RS3 alleles (one on each chromosome), and each is a certain length that falls within those ranges.

Variation in RS3 allele length has been linked to several important social processes in humans. For example, there is some evidence that variation in RS3 allele length is associated with social behaviors in sibling relationships and general concerns about the appropriateness of one’s social behavior (Bachner-Melman et al., 2005). Likewise, other research suggests that people who have two alleles of relatively shorter length were less likely to behave prosocially during an economic game compared to those with two relatively longer alleles (Knafo et al., 2008). Moreover, other research suggests that a *specific length* of an allele repeat increases people’s vulnerability to close relationship difficulties. For example, people with at least one allele with the repeat length of 327 base pairs (compared to people without an allele of this length) demonstrated lower cognitive empathy (Uzefovsky et al., 2015), suggesting that people with at least one copy of allele 327 have more difficulties recognizing others’ emotions. More directly related to close relationships, mothers who had at least one copy of allele 327 (compared to mothers without an allele of this length) were less likely to engage in supportive behavior with their child during a play session (Avinun et al., 2012). Taken together, these findings suggest that variation on RS3 has implications for social cognitions and behaviors that could influence relationship maintenance processes.

Nevertheless, very few studies have directly examined RS3 in the context of human pair bonding. Based on some evidence suggesting a link between RS3 and men’s sociosexual behavior (Prichard et al., 2007), Walum et al. (2008) used a sample of people in established relationships to examine whether variation on RS3 was associated with pair bonding difficulties in men. Of note, Walum et al. (2008) used a different set of primers than the research described above—resulting in different observed ranges of repeat lengths—but also found that a specific allele length was associated with men’s (but not women’s) vulnerability to pair bond difficulties. Specifically, men with at least one allele with the repeat length of 334 base pairs (compared to men without an allele of this length) were less likely to be married, more likely to experience marital crisis if they were married, and showed less “partner bonding” (a measure derived from pair bonding behaviors in non-human primates).

More recently, Acevedo et al. (2019) examined the association between RS3 and relationship outcomes in an in-depth fMRI study of newlywed participants (*n* = 18). In this case, RS3 variation was operationalized as having shorter or longer repeat lengths (relative to the median sample length). Although Acevedo et al. (2019, 2020) did not report the primers they used, the target allele (327 or 334) would likely have been categorized as a short allele given the sample median was reported as 335.86 base pairs. Consistent with Walum et al. (2008), the number of shorter alleles was negatively associated with relationship satisfaction and modulated participants’ neural activation in response to emotionally evocative images of their partner (but not of strangers). Inconsistent with Walum et al. (2008), this association was not moderated by sex. Further, analyses of 13 of the people who attended a second follow-up session implicated RS3 in maintaining romantic love over the first year of marriage (Acevedo et al., 2020). Overall, these two sets of findings appear consistent and together suggest that variation in RS3 allele length may be associated with difficulties in maintaining high levels of marital satisfaction.

Difficulties in maintaining high levels of marital satisfaction may reflect an orientation toward a short-term (vs. long-term) mating strategy (Buss and Schmitt, 1993). A short-term strategy orients people toward seeking a greater number of sexual partners and limiting investment in each romantic relationship. Especially for men, pursuing a short-term mating strategy may be one way to increase their reproductive success. Consistent with this theoretical account, Walum et al. (2008) found that men with at least one allele with the repeat length of 334 base pairs were less interested in commitment and reported more difficulties in committed relationships compared to men with no copies of this allele. Thus, variation in RS3 allele length may be one genetic marker of a predisposition toward a short-term mating strategy. Notably, variation on another repeat length polymorphism (i.e., CAG repeat length of the androgen receptor gene) has been argued to calibrate men’s physiological and psychological processes toward expending more effort in pursuit of mating (Roney et al., 2010; Simmons and Roney, 2011).

### 1.2 Current research

The aim of this research was to extend prior work by Walum et al. (2008) to examine whether the target *AVPR1A* RS3 allele 334 is associated with increased vulnerability to relationship difficulties and lower levels of relationship satisfaction over the first 3 years of marriage. We used data from a longitudinal study of newlywed couples to examine the extent to which *AVPR1A* RS3 allele 334 was associated with relationship maintenance processes and outcomes over the first 3 years of marriage. Given past research linking the presence of at least one copy of the target allele 334 to a lower likelihood of being married, we predicted that allele 334 would be negatively associated with indicators of relationship commitment. Specifically, we predicted that allele 334 would be negatively associated with relationship dedication, positively associated with desire for independence (as indexed by higher attachment avoidance), and positively associated with interest in romantic alternatives (i.e., potential romantic partners other than one’s current partner). Furthermore, given the link between allele 334 and increased reports of experiencing marital crises, we predicted that individuals with allele 334 would report more problems in domains related to intimacy and pair bonding, but not necessarily other problems such as those related to career pursuits or substance use. These hypotheses are also consistent with an orientation toward a short-term mating strategy.

For these target allele analyses, we focused on differences between individuals who had at least one copy of allele 334 and individuals without any copies of this target allele. Additionally, to strengthen conclusions about the unique importance of allele 334, we conducted ancillary analyses focusing on other frequent repeat lengths as potential target alleles, using the same *n* > 10 criteria as Walum et al. (2008).

We also operationalized RS3 allele length variation in a way similar to Acevedo et al. (2019, 2020). Because Acevedo et al. (2019, 2020) found that shorter alleles were associated with worse relationship outcomes, and because the target allele 334 was likely categorized as a short allele, we predicted that having more short alleles would be negatively associated with relationship dedication and positively associated with attachment avoidance, interest in romantic alternatives, and problems in domains related to intimacy and pair bonding. Examining both operationalizations increased the rigor of our methodological approach by allowing us to adjudicate between different types of associations RS3 repeat length may have with pair bonding in humans. Procyshyn et al. (2017) examined the link between RS3 and non-clinical presentations of autism-like traits using both operationalizations and found more support for a target allele approach.

In both sets of analyses, we explored whether associations were moderated by sex. Although Walum et al. (2008) found that RS3 was related to partner bonding for men but not women, Acevedo et al. (2019) did not find sex differences in the associations they observed, though they may have lacked the power to detect any differences that did exist due to their relatively small sample. Inconsistencies regarding whether associations between RS3 and human social behavior are moderated by sex are also documented outside of the research on romantic relationship processes. For example, Knafo et al. (2008) did not find that sex moderated the link between RS3 and prosocial behavior during an economic game. Moreover, Procyshyn et al. (2017) found an association between RS3 and non-clinical presentations of autism like traits for women but not men. Accordingly, we did not have strong predictions for the moderation.

Finally, in addition to testing the specific predictions outlined above, we conducted a series of exploratory analyses. First, to make use of this rich dataset, we explored associations with other processes relevant to pair bonding that were included in the baseline questionnaires couples completed at the start of marriage: trust, gratitude, forgiveness, jealousy, sexual satisfaction, sexual frequency, relationship attributions, oppositional behavior during conflict discussions, automatic partner attitudes, and automatic attention to romantic alternatives. Second, we explored whether variation on RS3 was associated with relationship outcomes by examining the trajectories of relationship satisfaction over the first 3 years of marriage.

## 2 Method

### 2.1 Participants

The study participants included 128 individuals (65 wives and 63 husbands) who were members of 70 heterosexual couples taking part in a larger, longitudinal study of new marriages. Analyses reported in this manuscript use a subsample of participants for whom samples were available for DNA analyses. The larger sample contained 240 members of 120 couples and has been described in prior research (McNulty et al., 2018). Couples were recruited using flyers and Facebook advertising targeting engaged and recently married couples. Eligibility required the couples be married less than 3 months. In general, participants were in their early 30s (Husbands: *M*_age_ = 32.20, *SD* = 10.98, range: 20–72; Wives: *M*_age_ = 30.33, *SD* = 8.48, range: 21–55). The majority (74.3%) of participants reported being White/Caucasian, the current marriage being their first marriage (77.1%), and not having children (75.0%). The study procedures were approved by the Florida State University Institutional Review Board (IRB) and research was conducted in accordance with the relevant guidelines and regulations set forth in the Belmont Report of the National Commission for the Protection of Human Subjects of Biomedical and Behavioral Research in 1979 as well as the American Psychological Association. All participants provided written informed consent to participate in the study.

The same sample and broad set of dependent variables were used in a previous paper that examined effects of variability on the SNP rs3796863 on *CD38* (Makhanova et al., 2021). All reported results are robust to the inclusion of rs3796863 as a covariate.

### 2.2 Procedure

Interested participants completed a telephone eligibility screening. Those couples who were eligible were then sent the baseline questionnaires before attending the lab session, which was scheduled within 3 months of the wedding. Couples were instructed to complete the measures individually. As part of the lab session, couples completed multiple different tasks, including those used in exploratory analyses (i.e., reaction time tasks assessing automatic partner attitudes and attention to romantic alternatives, as well as four problem-solving discussions). After the lab session, participants completed a short online survey every evening for 14 nights. For the longitudinal assessments, participants completed questionnaires every 4 months for the three-year study period. For the exploratory analyses examining longitudinal effects of RS3, we focused on participants’ reported marital satisfaction at each assessment (aggregated across three measures). Each assessment included the three measures of marital satisfaction that we standardized and used in exploratory analyses to examine RS3’s longitudinal effects.

### 2.3 Materials

The broader aim of the present research was to conduct comprehensive analyses examining links between RS3 repeat length variability and pair bonding. The first two authors selected measures from the larger study that would be most relevant to RS3 based on *a priori* predictions. We additionally identified other measures, less likely to be relevant to RS3 but relevant for relationship maintenance, for exploratory analyses.

#### 2.3.1 Primary dependent measures

##### 2.3.1.1 Marital satisfaction

At each assessment (every 4 months for 3 years), participants were asked to rate their marital satisfaction on three different measures: the Quality of Marriage Index (Norton, 1983), the Semantic Differential (Osgood et al., 1957), and the Kansas Marriage Satisfaction scale (Schumm et al., 1986). On average, participants were highly satisfied at baseline (Quality of Marriage Index: *M* = 42.03, *SD* = 4.95, range: 9 to 45; Semantic Differential: *M* = 95.69, *SD* = 10.80, range: 44 to 105; Kansas Marriage Satisfaction: *M* = 19.28, *SD* = 2.13, range: 9 to 21). All three were highly correlated (all *r*’s > 0.887). For the purposes of the present data analyses, we standardized each scale (by creating a Z-score) and created a composite, which was also standardized. For target allele analyses, we predicted that participants with at least one copy of allele 334 would report lower marital satisfaction than participants with no copies of allele 334. For the short/long categorization analyses, we predicted that the number of relatively shorter alleles would be negatively associated with marital satisfaction.

##### 2.3.1.2 Perceived severity of problems

Participants completed the Inventory of Marital Problems, which assesses problems couples face in their relationship (Geiss and O’Leary, 1981). Using a scale of 1 (Not a Problem) to 11 (A Major Problem) participants rated 19 common marital problems based on how problematic they were for their marriage. Problems were separated into three clusters: problems specific to pair bonding (7 problems: showing affection, amount of time spent together, recreation and leisure time, sex, trust, jealousy, and communication; *M* = 2.65, *SD* = 1.43, range: 1.00 to 8.86), problems related to other social relationships (3 problems: children; in-laws, parents, and relatives; friends; *M* = 2.62, *SD* = 1.46, range: 1.00 to 9.67), and problems not related to social relationships (9 problems: religion, household management, making decisions, unrealistic expectations, independence, money management, solving problems, drugs and alcohol, career decisions; *M* = 2.53, *SD* = 1.45, range: 1.00 to 8.22). For target allele analyses, we predicted that participants with at least one copy of allele 334 would report more problems related to pair bonding than participants with no copies of allele 334. For the short/long categorization analyses, we predicted that the number of relatively shorter alleles would be positively associated with problems related to pair bonding. In exploratory analyses, we examined whether RS3 variability was associated with the other two clusters of marital problems, as well as the composite of all marital problems.

##### 2.3.1.3 Noticing alternatives on a daily basis

During each daily diary survey over the course of the 14-day diary period, participants indicated using a scale of 1 (Not at all) to 7 (Very much) whether they noticed any romantic alternatives (someone of the opposite sex other than one’s partner). For analyses, we used an average of individuals’ responses across all completed diary surveys (*M* = 2.08, *SD* = 1.36, range: 1.00 to 6.46). For target allele analyses, we predicted that participants with at least one copy of allele 334 would report that they noticed more alternatives than participants with no copies of allele 334. For the short/long categorization analyses, we predicted that the number of relatively shorter alleles would be positively associated with noticing more alternatives.

##### 2.3.1.4 Attachment insecurity

Participants completed the Experiences in Close Relationships Scale—Revised (Fraley et al., 2010). Using a scale of 1 (Strongly Disagree) to 7 (Strongly Agree), participants indicated their agreement with statements assessing attachment anxiety (*n* = 9; *α* = 0.92; *M* = 2.22, *SD* = 0.99, range: 1.00 to 5.89; e.g., “I am afraid to lose my partner’s love”) and attachment avoidance (*n* = 9; *α* = 0.93; *M* = 2.23, *SD* = 0.92, range: 1.00 to 5.44; e.g., “I prefer not to show a partner how I feel deep down”). For target allele analyses, we predicted that participants with at least one copy of allele 334 would report greater attachment avoidance than participants with no copies of allele 334. For the short/long categorization analyses, we predicted that the number of relatively shorter alleles would be positively associated with attachment avoidance. In exploratory analyses, we examined whether RS3 variability was associated with attachment anxiety. Following other work in this literature (Chang and Overall, 2022), analyses involving each subscale controlled for the other subscale.

##### 2.3.1.5 Relationship dedication

We used two scales to assess relationship dedication. To assess general commitment, we used participants’ responses to the Commitment subscale of the Investment Model Scale (Rusbult et al., 1998) (*α* = 0.72; *M* = 8.13, *SD* = 0.77, range: 2 to 8.43; e.g., “I am committed to maintaining my relationship with my partner”). To assess specific dimensions of relationship dedication, we used participants’ responses to the Commitment Inventory (Stanley and Markman, 1992) which examines 10 different dimensions of commitment. Of the 10, we had predictions for six: relationship agenda (*α* = 0.71; *M* = 6.71, *SD* = 0.56, range: 2.83 to 7.00; e.g., “I want this relationship to stay strong no matter what rough times we may encounter”), prioritization of relationship (*α* = 0.75; *M* = 6.27, *SD* = 0.73, range: 3.17 to 7.00; e.g., “My relationship with my partner comes before my relationships with my friends”), satisfaction with sacrifice (*α* = 0.84; *M* = 5.71, *SD* = 0.97 range: 2.67 to 7.00; e.g., “It can be personally fulfilling to give up something for my partner”), couple identity (*α* = 0.76; *M* = 6.14, *SD* = 0.80, range: 3.83 to 7.00; e.g., “I like to think of my partner and me more in terms of ‘us’ and ‘we’ than ‘me’ and ‘him/her’”), and two dimensions focused on alternative romantic partners: availability of partners (*α* = 0.80, e.g., “It would be very difficult for me to find a new partner”) and alternative monitoring (*α* = 0.73, e.g., “I am not seriously attracted to members of the opposite sex other than my partner”). The latter two subscales (*α* = 0.74) were combined into one composite such that higher values indicated less desire for alternatives (*M* = 4.80, *SD* = 0.82, range: 2.55 to 6.75). We decided not to include the other four subscales because they focused on broader constraints (social pressure and structural investments subscales) and meta-cognitions about commitment (perceived morality of divorce and meta-commitment subscales). For target allele analyses, we predicted that participants with at least one copy of allele 334 would report lower relationship dedication than participants with no copies of allele 334. For the short/long categorization analyses, we predicted that the number of relatively shorter alleles would be negatively associated with relationship dedication.

#### 2.3.2 Exploratory analyses

We additionally explored whether RS3 repeat length variability was related to trust, gratitude, forgiveness, jealousy, sexual satisfaction, sexual frequency, relationship attributions, oppositional behavior during conflict discussions, automatic partner attitudes, and automatic attention to romantic alternatives. Please refer to the Supplementary material for more information about these measures.

### 2.4 Genotyping

Saliva samples were collected via passive drool and frozen at −20°C immediately after the lab session. Samples went through a freeze thaw cycle to remove the supernatant for hormone analyses. Pellets were refrozen and thawed for a second time for DNA purification. DNA was purified following a protocol adapted from past research (Beevers et al., 2009); 139 out of 142 samples provided adequate DNA concentrations. No issues with DNA integrity were observed in control analyses in which we tested 6 samples for DNA degradation on an agarose gel. The average yield was 494 ng/μL. In cases when the 260/280 ratio was below 1.80, we additionally ran the samples through a spin column from a Qiagen DNeasy Blood and Tissue kit following manufacturer instructions. DNA was diluted in TE buffer to a concentration of 28ng/μL prior to assaying.

Genotyping for RS3 followed the protocol outlined by previous research (Walum et al., 2008). RS3 repeat polymorphism was amplified with primers 5’-TCCTGTAGAGATGTAAGTGC-3′ (forward) and 5’-gtttcttTCTGGAAGAGACTTAGATGG-3′ (reverse) and read using the ABI PRISM 3730 Genetic Analyzer. The fragment lengths were analyzed using Peak Scanner Software v1.0. The software typically estimated lengths in decimals (e.g., 337.84) which were subsequently rounded up to the nearest whole even number (e.g., 338). We verified the distributions of the alleles by comparing them to those reported previously (Walum et al., 2008); allele 334 was also the most common allele in our sample. Allele frequencies are reported in Table 1. Because only 8 individuals (4 husbands and 4 wives all in different couples) were homozygous for allele 334, for our primary analyses we coded RS3 based on presence versus absence such that the absence of allele 334 was coded as 0 (*n* = 78) and the presence of at least one copy of allele 334 was coded as 1 (*n* = 51).

**Table 1:** Allele frequencies for RS3.

Allele	Husbands	Wives	Total
318	0	1	1
320	1	0	1
326	1	3	4
328	1	2	3
330	5	6	11
332	14	11	25
334	31	28	59
336	18	24	42
338	23	17	40
340	13	21	34
342	6	5	11
344	1	4	5
346	9	5	14
348	2	2	4
350	1	0	1
354	0	1	1

For analyses using the short/long categorization approach to allele parcellation, we conducted three sets of analyses, each using different cut offs for what allele was the last allele to be considered short. In two analyses, we relied on the central tendency measure in our own sample (*Mdn* = 336). We performed two sets of analyses: one in which allele 334 was the last allele categorized as short (i.e., allele 336 was the first allele categorized as long) and one in which allele 336 was the last allele categorized as short (i.e., allele 338 was the first allele categorized as long). Additionally, we conducted analyses in which allele 332 was the last allele to be categorized as short (i.e., allele 334 was the first allele categorized as long), following other studies that used the target allele as the starting point for long categorization (Knafo et al., 2008; Procyshyn et al., 2017). Allele frequencies for each of the three cut offs are presented in Supplementary Table S1 online.

### 2.5 Analytic strategy

To account for nesting of our dyadic data, we used multi-level modeling via the MIXED procedure in SPSS. For each operationalization of RS3 variability, our primary models predicted each dependent measure from RS3. Subsequent models examined whether the association between RS3 and the dependent measure was moderated by participant sex. Effect sizes are reported as *r*, calculated by first dividing the squared *t* value by the sum of the *t* value and the *df* from the model, and then taking the square root of the product (Rosenthal and Rosnow, 1991). All tests are two-tailed and use the alpha level of.05.

For the longitudinal analyses examining the trajectory of marital satisfaction, assessments were nested within each person, and time was crossed with dyad members. We used the MIXED command in SPSS to estimate a growth curve trajectory for each individual by regressing marital satisfaction onto a linear variable representing time (where the baseline assessment was coded as 0), RS3, and the RS3 x Time interaction. These models included random intercept and time effects for the husbands and wives. Model testing revealed that the best fitting model was one in which the intercepts were allowed to covary but the slopes were not. We also conducted a second set of analyses in which we estimated the same models, but with the time variable recentered such that the last assessment was coded as 0, thus making the simple effect of RS3 on marital satisfaction the association between RS3 and satisfaction at the end of the study (3 years after the baseline assessment).

## 3 Results

### 3.1 Target allele approach

First, we tested our prediction that the presence (versus absence) of allele 334 would be associated with lower relationship dedication and higher (i) attachment avoidance, (ii) interest in romantic alternatives, and (iii) problems in domains related to intimacy and pair bonding. As can be seen in Table 2, analyses did not support our predictions. In fact, several significant effects emerged that were in the direction opposite of our predictions. In contrast to the prediction that individuals with at least one copy of allele 334 would report more negative relationship processes than those with no copies of the allele, individuals with allele 334 perceived *fewer* problems in pair bond relevant areas of marriage and had *lower* desires for alternative partners compared to individuals without allele 334. A similar but non-significant trend emerged for marital satisfaction and the prioritization of the relationship. Notably, these associations were not moderated by participant sex. Allele 334 was not significantly associated with: (i) the other indices of relationship dedication (i.e., general commitment, having a strong relationship agenda, having a strong couple identity, or being satisfied with sacrificing for the relationship), (ii) noticing romantic alternatives during a daily diary component of the longitudinal study, or (iii) attachment avoidance.

**Table 2:** Associations between *AVPR1A* RS3 Allele 334 and relationship variables at the start of marriage.

	Association with Allele 334 (Absence = 0; Presence = 1)				
Dependent variable	*b*	*SE*	*t* (*df*)	*p*	*r*
Marital satisfaction	0.26	0.15	1.73 (86.50)	0.088	0.18
Problems (Pair bonding)	**−0.51**	**0.23**	**−2.22 (101.42)**	**0.029**	**0.22**
Noticing alternatives	−0.13	0.24	−0.56 (126.00)	0.577	0.05
Attachment avoidance	−0.24	0.15	−1.61 (125.00)	0.109	0.14
Relationship dedication					
General Commitment	−0.05	0.14	−0.32 (125.94)	0.748	0.03
Relationship agenda	−0.01	0.10	−0.09 (125.88)	0.930	0.01
Prioritization of relationship	0.21	0.13	1.67 (125.76)	0.098	0.15
Satisfaction with sacrifice	−0.10	0.18	−0.57 (126.00)	0.572	0.05
Couple identity	−0.05	0.14	−0.35 (123.39)	0.729	0.03
No desire for alternatives	**0.30**	**0.15**	**2.03 (125.99)**	**0.044**	**0.18**

Bolded effects are significant at p < 0.05. These are the results for the predicted associations. None of the effects were moderated by sex (all p’s > 0.099). See Supplementary materials for analyses controlling for participant race/ethnicity and prior history of marriage.

We next examined whether effects were due to having at least one allele with the specific repeat length of 334 base pairs versus other repeat lengths. We used the same approach as Walum et al. (2008) to examine seven other relatively common alleles (*n* > 10): 330, 332, 336, 338, 340, 342, and 346. We focused on people’s perceptions of relationship problems relating to pair bonding and intimacy because this variable most closely corresponds to the “Partner Bonding Scale” that Walum et al. (2008) used for the same analyses. As can be seen in Table 3, none of the seven other frequent alleles were associated with perceptions of problems relevant to pair bonding. These findings provide support for the specific importance of allele 334 (using these primers) in human pair bonding processes.

**Table 3:** Associations between the presence of common repeat lengths and problems with intimacy.

Allele	Freq.	*t*	*df*	*p*	*r*
330	11	−1.25	111.97	0.214	0.12
332	25	−0.04	105.94	0.969	0.00
334	59	**−2.22**	**101.42**	**0.029**	**0.22**
336	42	0.38	101.50	0.703	0.04
338	40	−0.47	109.81	0.637	0.04
340	37	0.14	109.78	0.892	0.01
342	11	−0.02	112.55	0.985	<0.01
346	14	0.10	102.82	0.919	0.01

Bolded effects are significant at *p* < 0.05. None of the effects were moderated by sex, all *p*’s > 0.114. Alleles that occurred at least 10 times were included in these analyses.

### 3.2 Short/long categorization approach

We then tested the primary hypotheses using the other operationalization of RS3 variability: categorizing alleles as either short or long. We analyzed the data using three different cutoff points for which allele was the last repeat length to be categorized as short: 336, 334, or 332. Following the pattern of findings reported by Acevedo et al. (2019), we predicted that the number of short alleles would be negatively associated with relationship dedication and positively associated with attachment avoidance, interest in romantic alternatives, and problems in domains related to intimacy and pair bonding. See Table 4 for results showing all three cutoff points for the short/long categorization.

**Table 4:** Associations between *AVPR1A* RS3 and relationship variables at the start of marriage.

Dependent variable	*b*	*SE*	*t* (*df*)	*p*	*r*
Allele 332 last categorized as short					
Marital satisfaction	−0.13	0.13	−1.04 (83.62)	0.301	0.11
Problems (Pair bonding)	0.08	0.20	0.39 (104.33)	0.700	0.04
Noticing alternatives	0.27	0.20	1.33 (126.00)	0.185	0.12
Attachment avoidance	0.22	0.14	1.53 (123.53)	0.128	0.14
Relationship dedication					
General commitment	0.05	0.12	0.39 (125.95)	0.695	0.03
Relationship agenda	0.02	0.09	0.27 (125.94)	0.789	0.02
Prioritization of relationship	0.14	0.11	1.30 (125.27)	0.195	0.12
Satisfaction with sacrifice	0.14	0.15	0.91 (126.00)	0.367	0.08
Couple identity	0.11	0.12	0.92 (123.32)	0.360	0.08
Low desire for alternatives x Sex	**−0.62**	**0.24**	**−2.60 (120.17)**	**0.011**	**0.23**
Simple Effect: Husbands	**−0.53**	**0.17**	**−3.14 (120.90)**	**0.002**	**0.27**
Simple Effect: Wives	0.10	0.17	0.56 (120.93)	0.576	0.05
Allele 334 Last categorized as short					
Marital satisfaction	0.05	0.10	0.46 (85.71)	0.649	0.05
Problems (Pair bonding)	−0.23	0.16	−1.45 (103.42)	0.150	0.14
Noticing alternatives	0.11	0.16	0.67 (126.00)	0.502	0.06
Attachment avoidance	0.01	0.12	0.11 (123.72)	0.910	0.01
Relationship dedication					
General commitment	0.04	0.10	0.45 (126.00)	0.655	0.04
Relationship agenda	0.03	0.07	0.48 (126.00)	0.631	0.04
Prioritization of relationship	**0.22**	**0.09**	**2.56 (124.86)**	**0.012**	**0.22**
Satisfaction with sacrifice	0.01	0.12	0.09 (126.00)	0.931	0.01
Couple identity	0.11	0.09	1.13 (123.05)	0.260	0.10
No desire for alternatives	0.02	0.10	0.24 (125.20)	0.811	0.02
Allele 336 last categorized as short					
Marital satisfaction	0.20	0.11	1.88 (83.27)	0.064	0.20
Problems (Pair bonding)	−0.14	0.17	−0.84 (102.28)	0.403	0.08
Noticing alternatives	−0.08	0.17	−0.49 (126.00)	0.628	0.04
Attachment avoidance	−0.02	0.12	−0.15 (122.75)	0.879	0.01
Relationship dedication					
General commitment	0.17	0.10	1.64 (126.00)	0.104	0.14
Relationship agenda	**0.16**	**0.07**	**2.12 (126.00)**	**0.036**	**0.19**
Prioritization of relationship	**0.26**	**0.09**	**2.95 (124.54)**	**0.004**	**0.26**
Satisfaction with sacrifice	0.14	0.13	1.09 (126.00)	0.278	0.10
Couple identity	0.15	0.10	1.49 (121.28)	0.139	0.13
No desire for alternatives	0.12	0.11	1.09 (124.72)	0.276	0.10

In these models, relationship measures are predicted from the number of short alleles (0 to 2). Bolded effects are significant at *p* < 0.05. Except for the desire for alternatives analyses when allele 332 was the last allele categorized as short, none of the effects were moderated by sex, all *p*’s > 0.073.

When allele 336 was categorized as the last short allele, results were once again opposite to our predictions. The number of short alleles was *positively* associated with two indicators of relationship dedication: prioritization of the relationship and relationship agenda (the aspect of commitment that reflects wanting to grow old with one’s spouse). There was also a non-significant trend for the number of short alleles being *positively* associated with marital satisfaction. No associations emerged between the number of short alleles and problems in domains related to intimacy and pair bonding, noticing alternatives, attachment avoidance, or the other four indices of relationship dedication (general commitment, satisfaction with sacrifice, couple identity, and desire for alternatives). When allele 334 was categorized as the last short allele, the only association that continued to emerge was prioritization of the relationship. When allele 332 was categorized as the last short allele, there were no significant main effects of RS3 for our primary dependent variables. However, the association between RS3 and desire for alternatives was moderated by sex. Although there was no association between RS3 and wives’ desire for alternatives, there was a negative association between the number of short alleles and husbands’ reporting that they do not desire alternative partners. That is, husbands who had more short alleles were more interested in romantic alternatives than husbands with fewer short alleles. Although this pattern is consistent with our original predictions, we hesitate to put emphasis on these findings considering they only emerged when the target allele was categorized as a long allele.

Overall, these results differed from those observed by Acevedo et al. (2019, 2020). That research found that the number of shorter alleles was negatively associated with pair bonding and relationship maintenance processes in the newlywed stage. In contrast, although several of the associations that emerged in our research were not significant, the ones that were significant suggested that the number of shorter alleles was *positively* associated with pair bonding and relationship maintenance processes.

### 3.3 Exploratory longitudinal analyses of marital satisfaction

**Figure 1:**
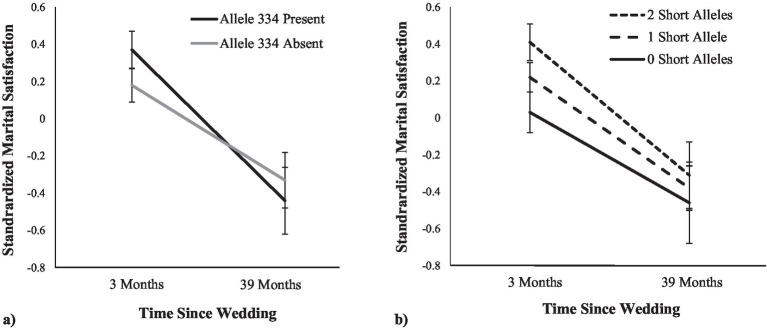
Associations between AVPR1a RS3 and marital satisfaction over the first 3 years of marriage. Panel **A** shows the effect with the focus on allele 334; Panel **B** shows the effect with the focus on the number of short alleles (with allele 336 as the last allele categorized as short). Error bars are SE.

Next, we examined the association between variability on RS3 repeat length and satisfaction over time. Of note, as in the baseline analyses, none of the effects differed across men and women and so all parameter estimates were pooled across husbands and wives. First, we used the target allele approach and examined how baseline marital satisfaction and the trajectory of marital satisfaction differed between people who had at least one copy of allele 334 and those without any alleles of this length (see Figure 1A). Similar to the baseline analyses reported in Table 2, there was a non-significant trend for allele 334 to be positively associated with marital satisfaction at baseline, *b* = 0.09, *SE* = 0.05, *t* (106.16) = 1.79, *p* = 0.076. Consistent with past research, there was a main effect of time such that participants reported lower marital satisfaction over time, *b* = −0.07, *SE* = 0.01, *t* (101.83) = −5.52, *p* < 0.001. Although participants who had at least one copy of allele 334 did not differ from those with no copies of allele 334 in the extent to which their marital satisfaction changed over time, *b* = −0.02, *SE* = 0.01, *t* (86.69) = −1.38, *p* = 0.172, the analysis that re-centered the time variable such that the intercept represented the end-point of the study revealed that individuals with allele 334 were not more or less satisfied than individuals without allele 334, *b* = −0.06, *SE* = 0.11, *t* (88.34) = −0.52, *p* = 0.603. In other words, the trend for initial differences appeared to wear off over time.

We then performed the same analyses using the short/long categorization approach. For the number of short alleles, when allele 336 was categorized as the last short allele, we found the same pattern (see Figure 1B). At the baseline assessment, the number of short alleles was *positively* associated with marital satisfaction, *b* = 0.13, *SE* = 0.05, *t* (97.57) = 2.66, *p* = 0.009. Again, there was a main effect such that participants reported lower marital satisfaction over time, *b* = −0.07, *SE* = 0.01, *t* (101.49) = −5.49, *p* < 0.001. As in the target allele analyses, the number of short alleles did not moderate the trajectory of marital satisfaction, *b* = −0.01, *SE* = 0.01, *t* (83.17) = −0.74, *p* = 0.464, but the analysis that re-centered the time variable to the end-point of the study revealed that the number of short alleles was not associated with marital satisfaction, *b* = 0.05, *SE* = 0.11, *t* (82.43) = 0.47, *p* = 0.639. When allele 334 was categorized as the last short allele, we found a similar pattern, but with no significant results (baseline assessment: *b* = 0.08, *SE* = 0.05, *t* [102.16] = 1.61, *p* = 0.110; interaction: *b* = −0.01, *SE* = 0.01, *t* [83.40] = −0.73, *p* = 0.465; end-point assessment, *b* = 0.003, *SE* = 0.11, *t* [85.01] = 0.03, *p* = 0.974). Finally, when allele 332 was categorized as the last short allele, we again found no significant results (baseline assessment: *b* = −0.01, *SE* = 0.05, *t* [98.36] = 0.17, *p* = 0.863; interaction: *b* = 0.003, *SE* = 0.01, *t* [83.08] = 0.28, *p* = 0.783; end-point assessment: *b* = 0.04, *SE* = 0.11, *t* [85.25] = 0.36, *p* = 0.723).

### 3.4 Exploratory analyses for relationship maintenance processes

Finally, we conducted exploratory analyses examining other relationship maintenance processes that were assessed as part of the longitudinal study. The full descriptions of the measures and results are presented as Supplementary material. When we conducted analyses using the target allele approach (see Supplementary Table S2), we found that individuals with allele 334 reported fewer problems than those without the allele across *all* types of domains, *b* = −0.52, *SE* = 0.21, *t* (97.88) = −2.47, *p* = 0.015, *r* = 0.24, not just those relevant to intimacy and pair bonding. No other exploratory relationship maintenance processes were associated with allele 334.

When we repeated these analyses using the short/long categorization approach (see Supplementary Table S3), where allele 336 was categorized as the last short allele, we found that the number of short alleles was negatively associated with behavioral jealousy, *b* = −0.26, *SE* = 0.12, *t* (125.10) = −2.25, *p* = 0.026, *r* = 0.20, which is the subscale of the Multidimensional Jealousy Scale (Pfeiffer and Wong, 1989) that is associated with behaviors such as looking through partners’ possessions for signs of cheating. When either allele 334 or allele 332 was categorized as the last short allele, no significant associations emerged between the number of short alleles and relationship maintenance processes.

## 4 Discussion

We examined whether variations in the repeat polymorphism RS3 on the *AVPR1A* gene were associated with relationship processes and outcomes in newlywed couples. We tested our hypotheses using two operationalization approaches common to the literature—whether the presence of target allele 334 and the number of shorter alleles would have been negatively associated with relationship maintenance processes. Prior research has linked the presence of allele 334 to lower likelihood of marriage and, if married, greater reports of marital crises among men (Walum et al., 2008). Other research has linked the number of shorter repeat lengths (with allele 334 seeming to be categorized as a short allele) to lower relationship satisfaction (Acevedo et al., 2019). The present findings, although preliminary in light of our small sample size, did not appear to replicate either pattern and did not suggest that variation on RS3 uniformly predisposes people to adopt a short-term mating strategy.

In contrast, when associations with RS3 variation were significant in our analyses, the associations were in the opposite direction, using both analytic approaches. The target allele analyses found preliminary evidence that husbands and wives with allele 334 reported fewer problems in all domains of their marriage, lower desire for alternative partners, and trended toward being more satisfied with their relationships at the time of marriage. Similarly, the number of shorter alleles tended to be associated with greater marital satisfaction at the start of marriage and desire to grow old with one’s spouse. In the longitudinal analyses, however, we no longer saw differences among RS3 genotypes in marital satisfaction at the three-year assessment.

Further, although some prior research suggests that RS3 has stronger implications for men’s pair bonding than women’s (Walum et al., 2008), we did not observe sex differences in our data in analyses using either operationalization. It could be that the current study lacked power to detect this sex difference. Indeed, the twin sample used by Walum et al. (2008) included 1899 participants (810 men) whereas our sample had 128 participants (63 men). Alternatively, it may be that for certain processes, RS3 has analogous effects for men and women who decide to get married. Other research has similarly found no sex differences (Knafo et al., 2008).

### 4.1 Reconciling findings with past research

One potential explanation for the differences between our preliminary findings and those reported by Walum et al. (2008) is that the relational phase is critical to understanding the role of RS3 in couple functioning. Because of the inclusion criteria for the twin sample used by Walum et al. (2008), participants were married or cohabitating for at least 5 years and were parents of an adolescent child (average age of 15 years old). Conversely, our sample consisted entirely of recently married couples. The average premarital relationship length was 4 years (*M* = 49.51 months, *SD* = 35.74, range: 2 to 206 months) and only 25% of participants had children. In other words, the participants in the two samples were at very different relational phases. Thus, both sets of findings could be true, depending on the phase of the relationship being considered; it may be that people with allele 334 experience enhanced passion and satisfaction at the beginning of their relationships relative to those without the allele (as observed in our study), but that such individuals also lose interest more quickly, eventually coming to rest in their relationships as less satisfied and more conflictual (as observed by Walum et al., 2008). Consistent with this possibility, although there was a trend for our participants with allele 334 to be more satisfied in the newlywed stage, they were not more satisfied 3 years into marriage. In fact, visual inspection of the data suggests that they had steeper declines in marital satisfaction during the first 3 years of marriage compared to those without allele 334. Had we followed these couples for a longer amount of time, we may have observed that individuals with allele 334 would have become less satisfied than those without allele 334. Such a pattern of responding—higher highs at the beginning of the relationship and lower lows as time goes on—is consistent with the broader possibility that individuals with allele 334 may generate more relationship discord and may even be more likely to engage in serial monogamy. That is, when satisfaction with their current relationship wanes, a new partner may ignite strong feelings that make people more likely to move on to the new relationship. Future research may benefit from addressing this possibility directly.

Another potential explanation for the observed differences, which also stems from the timing of our assessment, is cognitive dissonance. Cognitive dissonance is a motivated state that emerges when people’s behavior and attitudes are inconsistent; when such inconsistencies arise, the aversive experience of cognitive dissonance prompts people to resolve the inconsistency by either changing their behavior or their attitudes (Cooper, 2019; Festinger and Carlsmith, 1959). Because behavior is difficult to change (as it has already occurred), people typically resolve dissonance by changing their attitudes (often in ways that conflict with core beliefs). In the context of the present research, cognitive dissonance may lead participants with at least one copy of allele 334 to report higher marital satisfaction and lower relationship problems. Specifically, Walum et al. (2008) found that those (men) with at least one copy of allele 334 were less likely to be married, but we found that those with at least one copy of allele 334 reported higher levels of marital satisfaction. If someone is hesitant to commit to a relationship, but they end up taking the highly committed steps of getting a marriage license, finding an officiant, and submitting the paperwork that legally and publicly ties them to their romantic partner, the inconsistency between their attitudes (generally non-committal) and behavior (just got married) may lead to cognitive dissonance. Because people are motivated to alleviate dissonance, and because the behavior has already occurred (they are married), cognitive dissonance may drive people to change their attitudes and conclude that they would have only made such a commitment if their partner and relationship are of exceptionally high quality. This process could prompt people to self-report particularly high levels of relationship satisfaction and low levels of relationship problems. Of course, people are unlikely to be able to maintain any illusions over longer periods of time, and thus any initial boost in satisfaction may wane over time, as appeared to be the case here. Future research would benefit from directly examining whether pre-marital hesitation to commit leads to cognitive dissonance, which may in turn affect the association between relationship satisfaction and RS3.

Finally, although we suspect this explanation is less likely, the difference may reflect the fact that either our results or both our results and those reported by Walum et al. (2008) are Type I errors. We doubt this is the case for the prior studies because the same basic finding emerged across two similar samples. We doubt this is the case for our study because the same pattern emerged across a host of outcomes. Still, it is always possible that “file drawers” contain numerous studies that observed no association between variation in RS3 and relationship processes that have never been published.

### 4.2 Addressing methodological inconsistencies

All this said, direct comparison between studies is complicated by how variability in RS3 is operationalized. Two approaches are typical: categorizing alleles as short or long (e.g., Acevedo et al., 2019; Acevedo et al., 2020; Knafo et al., 2008) or focusing on effects of a target allele (e.g., allele 334 for one set of primers, Walum et al., 2008; allele 327 for another set of primers, Uzefovsky et al., 2015). The choice of which approach to take could be somewhat arbitrary. That is, studies select one approach and not the other without providing a rationale for their choice. Only one other study to our knowledge conducted and reported results using both approaches (Procyshyn et al., 2017). Those findings provided support for the specific importance of a target allele (allele 330 with the set of primers used in that research) for non-clinical presentations of autism-like traits in women.

It may be important for researchers to be more deliberate in their choice of approach because each approach implies different functional roles for the RS3 polymorphism. Associations between repeat length polymorphisms and behavior can take different forms, such as linear, curvilinear, threshold, or stochastic. Examining differences between shorter and longer repeat lengths assumes a threshold relationship between repeat length and a quantitative trait. However, examining effects of a specific target allele assumes a stochastic relationship, such that only a specific length acts differently from other lengths. With respect to our study, on the one hand, and consistent with Walum et al. (2008), our findings are generally supportive of the specific role of target allele 334 in perceptions of marital problems and a stochastic relationship between RS3 repeat length and human social behavior. In this light, our findings are consistent with past studies showing vulnerabilities to social difficulties conferred by a specific repeat length (Walum et al., 2008; Uzefovsky et al., 2015; Avinun et al., 2012; Procyshyn et al., 2017). On the other hand, some of our results are also consistent with the idea that there is a threshold relationship between RS3 repeat length and human social behavior, an idea that is implied by the decision to categorize repeat lengths as either short or long (Knafo et al., 2008; Acevedo et al., 2019; Acevedo et al., 2020).

Inconsistencies in the literature linking genes to sociosexual processes rightfully increase wariness of findings from candidate gene studies. One way to strengthen conclusions about effects of a polymorphism on a behavioral phenotype is to conduct genome-wide association studies (GWAS) (Landefeld et al., 2018). However, GWAS methods historically only apply to research focusing on SNPs and have not been applicable to repeat-length polymorphisms such as RS3. Emerging technologies may overcome this limitation and permit growth of GWAS studies with repeat polymorphisms in the near future (e.g., Mukamel et al., 2021; Ziaei Jam et al., 2023). Nevertheless, these future GWAS studies will also have to contend with differences in operationalizations of genotypes (i.e., target repeat length vs. short/long categorization). The rationale for operationalizations of repeat genotypes must be grounded in putative mechanisms, and may be locus-specific. Thus, candidate gene studies such as this one are critical for providing such grounding rationale by systematically examining whether the polymorphism demonstrates a threshold or a stochastic relationship with human pair bond maintenance. Furthermore, GWAS studies require samples in the tens of thousands (Benjamin et al., 2012; Chabris et al., 2015; Dick et al., 2015), and it would not be feasible to conduct an intensive longitudinal study of married couples using GWAS, from either a methodological or practical standpoint. It is our view that candidate gene and GWAS approaches both present limitations and opportunities and, ultimately, the best evidence will come from a triangulation of methods and transparent reporting of findings.

### 4.3 Limitations and future directions

Our study was not without limitations. First, we were only able to genotype 128 individuals from 70 newlywed couples, limiting our power to test the predicted associations, particularly for the interaction effects we examined in our exploratory analyses. Indeed, our post-hoc sensitivity analyses showed that we had 80% power to detect effect sizes of *r* of 0.23–0.30, whereas most of our observed effect sizes for statistically significant associations were between 0.18 and 0.22 (see Supplementary materials for sensitivity analyses for each dependent measure). Moreover, small sample sizes are a perennial challenge of candidate gene studies (Benjamin et al., 2012; Chabris et al., 2015; Dick et al., 2015). Although our sample size was much larger than one set of previous studies (Acevedo et al., 2019; Acevedo et al., 2020), as we noted, Walum et al. (2008) used a much larger data set. Of course, that study too had limitations, including the fact that it was not specifically designed to examine romantic relationships. Accordingly, future research may help reconcile some of the inconsistencies across existing studies and this one with well-powered research prospective designs specifically targeting relationship processes. A multi-lab collaboration is one way to achieve such a study, given the considerable labor and financial resources that it would entail.

Second, because our study was designed to examine romantic relationships, participants knew from the advertisements that the study would focus on their relationships. This explicit focus makes it possible that participants who were particularly unsatisfied with their relationships self-selected out of our study. Although the distribution of alleles in this sample was quite similar to that of other studies, we cannot rule out the possibility that self-selection restricted the range of participants with at least one copy of allele 334 and contributed to the pattern of findings observed in the present sample.

A third limitation is the fact that, across tests of our predictions, robustness checks including other operationalizations of RS3 variability, and exploratory analyses, we estimated numerous statistical models. Although readers should be cautious in interpreting individual effects, the fact that notable and consistent patterns emerged across several dependent variables gives us more confidence in the patterns themselves. Future research may benefit from conceptually replicating the specific effects.

Fourth, our sample lacked global genetic and cultural diversity given that participants were predominantly White. Our exploratory analyses examining whether race and ethnicity moderated the associations between the presence of allele 334 and relationship processes suggested that among non-White participants there was a trend for the predicted association (i.e., presence of allele 334 was marginally negatively associated with two measures of commitment). However, only a quarter of our (small) sample was non-White. Moreover, congruent with our sample demographics, we focused on Western cultural norms of romantic relationships and different patterns may emerge in non-Western samples.

Finally, although our findings join a body of literature that links polymorphisms in *AVPR1A* with predispositions toward different behavioral strategies in romantic relationships, the mechanism(s) underlying these phenotypes remains unknown. Emerging clues suggest that the RS3 polymorphism in *AVPR1A* can contribute to differences in gene expression in cultured cells (Tansey et al., 2011) and in the human brain (Landefeld et al., 2018; Knafo et al., 2008). It is not known how individual differences in *AVPR1A* impact specific brain networks acutely in adult relationship processes and/or potentially impact the development of brains that are or are not suited to these relationship maintenance processes.

At the same time, there are also several strengths of this research that should be weighed against these limitations. First, the data allowed for a rigorous investigation of the associations between RS3 and romantic relationship processes. Indeed, whereas many previous studies included only a few measures of relationship-relevant processes into broader studies of genetic influences on human behavior, we examined a broad range of processes that were specifically selected to examine relationship maintenance behavior. Moreover, the focus on the newlywed period has allowed us to uncover the possibility that the associations between RS3 and pair bonding may be moderated by relationship length.

## Data Availability

These data cannot be deposited in a public repository for ethical and privacy reasons because participants did not consent to have their data made publicly available and because couple member data may be identifiable to each other. We will, however, make the data available upon request for such purposes as confirming study results, conducting meta-analyses, etc. A Data Access Committee, consisting of Drs. Makhanova and McNulty will assess requests for access to the data. Inquiries should be sent to Dr. McNulty at: mcnulty@psy.fsu.edu.
