# Supplementary material for: AVPR1A RS3 and relationship maintenance processes in newlywed couples
Source: Front Psychol. 2025 Mar 3;16:1303065. doi: 10.3389/fpsyg.2025.1303065 (PMC11911472; doi:10.3389/fpsyg.2025.1303065)
Supplement: Supplementary file 1 [file Supplementary_file_1.docx]

*AVPR1A* RS3 and Relationship Maintenance Processes in Newlywed Couples

Supplementary Material

# Exploratory Measures

Trust. Participants answered three questions (α = .86; *M* = 6.56, *SD* = 0.74, range: 3.33 to 7.00) from the Trust subscale of the Perceived Relationship Quality Components Inventory (81) which asked participants to indicate their agreement with each statement (e.g., “How much do you trust your partner?”) on a scale of 1 (Not at all) to 7 (Completely) scale.

Gratitude*.* Participants answered six questions (α = .82; *M* = 4.78, *SD* = 0.34, range: 3.50 to 5.00) about their gratitude toward their partner (Lambert, et al., 2010). Participants were asked to indicate the frequency with which they typically feel and express gratitude for their partner (e.g., I feel appreciation for the things that my partner does for me”) on a scale of 1 (Never) to 5 (Frequently).

Forgiveness. Participants were asked to imagine their partner committing five hypothetical transgressions (McNulty, 2011) and then indicate the extent to which they would forgive their partner in each scenario on a scale of 1 (Definitely no) to 7 (Definitely yes) (*M* = 4.76, *SD* = 1.23, range: 1.60 to 7.00).

Jealousy. Participants completed the Multidimensional Jealousy Scale (Pfeiffer & Wong, 1989) which examines three different domains of jealousy: emotional (*n* = 8; α = .87; *M* = 4.50, *SD* = 0.88, range: 1.67 to 6.83; e.g., “Your spouse comments to you on how great looking a particular member of the opposite sex is”), cognitive (*n* = 7; α = .87; *M* = 1.70, *SD* = 0.88, range: 1.00 to 4.88; e.g., “I suspect that my spouse is secretly seeing someone of the opposite sex”), and behavioral (*n* = 8; α = .82; *M* = 1.85, *SD* = 0.88, range: 1.00 to 5.13; e.g., “I look through my spouse’s drawers, handbag, pockets, phone, or emails”). Participants indicated responses using 7-point scales.

Sexual Satisfaction. Participants completed the Index of Sexual Satisfaction (Hudson, 1998). Participants indicated their agreement with 25 statements (α = .93; *M* = 5.89, *SD* = 0.75, range: 3.20 to 6.96; e.g., “I think that our sex is wonderful”) on a scale from 1 (None of the time) to 7 (All of the time).

Sexual Frequency. Participants were asked the following open-ended question “Approximately how many times have you had sexual intercourse with your spouse over the past 4 months?” On average, participants reported 30 instances (*M* = 29.91, *SD* = 20.23; range: 0 to 100).

Attributions. To assess people’s tendencies to attribute their partner’s negative behavior to their partner, we asked participants to complete the Relationship Attribution Measure (Fincham & Bradbury, 1992) (*M* = 3.66, *SD* = 1.02; range: 1.46 to 6.13).

Oppositional Behavior. During the lab session, couples engaged in four problem-solving discussions. Video recordings of each discussion were coded based on speaking turns (Makhanova et al., 2018). A speaking turn was coded as oppositional if it contained one of three direct (blaming, rejecting, making demands of the partner) or one of three indirect (sarcasm, hostile joking, hostile questioning, mind-reading, denying responsibility) oppositional behaviors. For analyses, we computed the proportion of total speaking turns that were coded as oppositional and averaged the proportions across the four conversations (*M* = 0.08, *SD* = 0.10, range: 0.00 to 0.75).

Automatic Partner Attitudes. Participants completed the Partner Evaluative Priming Task that required participants to categorize words as either good or bad after seeing photos of their partner (analyses controlled for latencies for positive and negative primes in the practice blocks; McNulty et al., 2019). Below are the descriptive statistics for the subsample used in the present analyses.

Partner attitudes: *M* = 1.78, *SD* = 100.25, range: -228.68 to 251.44

Positive primes in practice blocks: *M* = 693.28, *SD* = 158.32, range: 460.07 to 1595.20

Negative primes in practice blocks: *M* = 740.94, *SD* = 172.84, range: 491.56 to 1499.36

Automatic Attention to Romantic Alternatives. Participants completed a dot probe attention task that assessed the latency with which participants categorized target shapes (circle or square) following the presentation of a face in a different quadrant of the screen (see Maner et al., 2007). We were interested in people’s automatic attention to romantic alternatives, thus our analyses focused on latencies following the presentation of attractive opposite-sex faces, while controlling for latencies following the presentation of the average opposite-sex targets, attractive same-sex faces, and average same-sex faces (McNulty et al., 2018). Below are the descriptive statistics for the subsample used in the present analyses.

Attractive Opposite Sex: *M* = 511.42, *SD* = 90.20, range: 332.50 to 835.42

Attractive Same Sex: *M* = 517.75, *SD* = 97.06, range: 351.42 to 854.50

Average Opposite Sex: *M* = 515.37, *SD* = 92.09, range: 333.55 to 900.00

Average Same Sex: *M* = 510.25, *SD* = 94.15, range: 357.22 to 916.09

# Supplementary Tables

Supplementary Table S1

*Allele frequencies for RS3 using short/long categorization*

| Allele | Husbands | Wives | Total |
| --- | --- | --- | --- |
| Alleles 318-332 Categorized as Short |  |  |  |
| 0 | 3 | 4 | 7 |
| 1 | 17 | 14 | 31 |
| 2 | 45 | 45 | 90 |
| Alleles 318-334 Categorized as Short |  |  |  |
| 0 | 9 | 14 | 23 |
| 1 | 33 | 25 | 58 |
| 2 | 23 | 24 | 47 |
| Alleles 318-336 Categorized as Short |  |  |  |
| 0 | 19 | 21 | 40 |
| 1 | 37 | 29 | 66 |
| 2 | 9 | 13 | 22 |

Supplementary Table S2

*Exploratory Analyses for AVPR1A RS3*

|  | Association with Allele 334  (Absence = 0; Presence = 1) | | | | |  |
| --- | --- | --- | --- | --- | --- | --- |
| Dependent Variable | *b* | *SE* | *t* (*df*) | *p* | *r* | |
| Problems in Other Social Areas | -0.43 | 0.25 | -1.74(111.35) | .084 | .16 | |
| Problems in Non-Social Areas | **-0.52** | **0.24** | **-2.21(100.33)** | **.030** | **.22** | |
| All Problems | **-0.52** | **0.21** | **-2.47(97.88)** | **.015** | **.24** | |
| Trust | 0.02 | 0.13 | 0.15(118.75) | .885 | .01 | |
| Gratitude | -0.01 | 0.06 | -0.20(115.45) | .842 | .02 | |
| Forgiveness | -0.11 | 0.22 | -0.50(119.60) | .621 | .05 | |
| Emotional Jealousy | 0.20 | 0.17 | 1.12(116.58) | .263 | .10 | |
| Cognitive Jealousy | 0.05 | 0.17 | 0.29(125.88) | .774 | .03 | |
| Behavioral Jealousy | -0.26 | 0.16 | -1.62(126.00) | .107 | .14 | |
| Attachment Anxiety | 0.22 | 0.16 | 1.35(125.00) | .180 | .12 | |
| Sexual Satisfaction | 0.03 | 0.12 | 0.29(101.53) | .773 | .03 | |
| Sexual Frequency | 2.42 | 3.00 | 0.81(63.89) | .424 | .10 | |
| Attributions | -0.14 | 0.19 | -0.73(125.98) | .465 | .06 | |
| Oppositional Behavior | 0.02 | 0.02 | 1.44(93.23) | .154 | .15 | |
| Automatic Partner Attitudes | -15.83 | 11.69 | -1.35(118.63) | .178 | .12 | |
| Automatic Attention to Alternatives | -2.74 | 6.76 | -0.41(121.00) | .686 | .04 | |

*Note.* Bolded effects are significant at *p* < .05. None of the effects were moderated by sex, all *p*’s > .102.

Supplementary Table S3

*Exploratory associations between the number of short alleles on RS3 and relationship variables at the start of marriage.*

|  | Allele 332 Last Categorized as Short (318-332) vs (336-354) | | | | | Allele 334 Last Categorized as Short  (318-334) vs (336-354) | | | | | Allele 336 Last Categorized as Short  (318-336) vs (338-354) | | | | |  |
| --- | --- | --- | --- | --- | --- | --- | --- | --- | --- | --- | --- | --- | --- | --- | --- | --- |
| Dependent Variable | *b* | *SE* | *t* (*df*) | *p* | *r* | *b* | *SE* | *t* (*df*) | *p* | *r* | *b* | *SE* | *t* (*df*) | *p* | *r* |  |
| Problems (Other Social) | 0.06 | 0.21 | 0.29(109.91) | .773 | .03 | -0.15 | 0.17 | -0.87(110.68) | .384 | .08 | -0.01 | 0.18 | -0.06(107.55) | .949 | .01 | |
| Problems (Non-Social) | 0.14 | 0.20 | 0.70(102.77) | .483 | .07 | -0.19 | 0.16 | -1.16(100.84) | .248 | .11 | -0.30 | 0.17 | -1.76(98.21) | .081 | .17 | |
| Problems (All) | 0.12 | 0.18 | 0.65(100.40) | .518 | .06 | -0.19 | 0.15 | -1.33(100.43) | .186 | .13 | -0.19 | 0.15 | -1.27(98.79) | .206 | .13 | |
| Trust | 0.07 | 0.11 | 0.65(117.21) | .520 | .06 | 0.06 | 0.09 | 0.62(116.27) | .534 | .06 | 0.13 | 0.09 | 1.41(114.38) | .163 | .13 | |
| Gratitude | -0.01 | 0.05 | -0.20(114.27) | .841 | .02 | 0.003 | 0.04 | 0.06(113.61) | .950 | .01 | -0.03 | 0.04 | -0.70(111.44) | .484 | .07 | |
| Forgiveness | -0.23 | 0.18 | -1.23(116.54) | .221 | .11 | -0.09 | 0.15 | -0.61(116.92) | .544 | .06 | -0.04 | 0.16 | -0.24(114.60) | .811 | .02 | |
| Emotional Jealousy | 0.10 | 0.15 | 0.67(114.04) | .505 | .06 | 0.13 | 0.12 | 1.09(113.13) | .278 | .10 | 0.12 | 0.12 | 0.99(110.54) | .322 | .09 | |
| Cognitive Jealousy | 0.004 | 0.14 | 0.03(125.71) | .976 | <.01 | 0.02 | 0.11 | 0.20(125.18) | .844 | .02 | -0.10 | 0.12 | -0.82(124.69) | .415 | .07 | |
| Behavioral Jealousy | -0.05 | 0.14 | -0.34(125.93) | .738 | .03 | -0.16 | 0.11 | -1.44(125.84) | .154 | .13 | **-0.26** | **0.12** | **-2.25(125.10)** | **.026** | **.20** | |
| Attachment Anxiety | -0.07 | 0.14 | -0.50(124.95) | .620 | .04 | 0.08 | 0.11 | 0.68(124.78) | .497 | .06 | 0.02 | 0.12 | 0.19(124.10) | .848 | .02 | |
| Sexual Satisfaction | -0.02 | 0.10 | -0.19(98.43) | .849 | .02 | 0.001 | 0.08 | 0.02(99.08) | .983 | <.01 | -0.04 | 0.08 | -0.47(96.52) | .637 | .05 | |
| Sexual Frequency | 0.56 | 2.46 | 0.23(65.19) | .820 | .03 | 1.13 | 2.00 | 0.57(60.76) | .574 | .07 | -1.18 | 2.17 | -0.54(65.71) | .588 | .07 | |
| Attributions | 0.02 | 0.16 | 0.14(126.00) | .888 | .01 | -0.06 | 0.13 | -0.44(125.69) | .659 | .04 | 0.01 | 0.14 | 0.08(125.30) | .938 | .01 | |
| Oppositional Behavior | -0.02 | 0.01 | -1.78(91.29) | .078 | .18 | -0.003 | 0.01 | -0.28(97.33) | .780 | .03 | -0.01 | 0.01 | -0.89(95.78) | .378 | .09 | |
| Auto. Partner Attitudes | -2.61 | 9.86 | -0.27(118.06) | .792 | .02 | -13.28 | 7.94 | -1.67(116.06) | .097 | .15 | -12.49 | 8.49 | -1.47(116.17) | .144 | .14 | |
| Auto. Attention to Alts | 5.64 | 5.78 | 0.98(121.00) | .331 | .09 | 3.66 | 4.75 | 0.77(121.00) | .443 | .07 | 1.01 | 4.95 | 0.20(121.00) | .839 | .02 | |

*Note.* Bolded effects are significant at *p* < .05. None of the effects were moderated by sex, all *p*’s > .070.

Supplementary Table S4

*Sensitivity Power Analyses*

|  |  |
| --- | --- |
| Dependent Variable | 80% to detect *r* effect size of |
| Confirmatory |  |
| Marital Satisfaction (cross-sectional) | .30 |
| Problems in Pair-Bonding | .28 |
| Noticing Alternatives | .23 |
| Attachment Avoidance | .24 |
| General Commitment | .23 |
| Relationship Agenda | .23 |
| Prioritization of Relationship | .24 |
| Satisfaction with Sacrifice | .24 |
| Couple Identity | .24 |
| No desire for alternatives | .24 |
| Exploratory |  |
| Marital Satisfaction (longitudinal) | .23 |
| Problems in Other Social Areas | .28 |
| Problems in Non-Social Areas | .28 |
| All Problems | .28 |
| Trust | .25 |
| Gratitude | .26 |
| Forgiveness | .25 |
| Emotional Jealousy | .27 |
| Cognitive Jealousy | .24 |
| Behavioral Jealousy | .23 |
| Attachment Anxiety | .24 |
| Sexual Satisfaction | .29 |
| Sexual Frequency | .25 |
| Attributions | .23 |
| Oppositional Behavior | .28 |
| Automatic Partner Attitudes | .24 |
| Automatic Attention to Alternatives | .24 |

# Supplementary Analyses

In additional analyses, we examined whether the association between the presence of allele 334 and our primary dependent variables were robust in analyses controlling for binary variables of participant race/ethnicity and prior history of marriage.

First, we conducted two chi square test of independence to determine whether the frequencies of allele 334 differed between these demographic groups. We did not find evidence for differences between people who self-identified as White/Caucasian (*n* = 104) and from people who self-identified as another race or ethnicity (*n* = 36) on the frequency of having the target allele, χ^2^(1) = 0.98, *p* = .322. Of course, a limitation of this approach is that we treated all non-White participants as part of the same group. However, we did not have the sample size for any race or ethnicity to conduct these analyses with more nuance. Twenty-two percent of our participants reported being married previously. Notably, there was no difference in frequencies of allele 334 carriers between the groups based on prior history of marriage, χ^2^(1) = 1.30, *p* = .254.

Next, we added the two binary covariates to the multi-level models we used to test our primary predictions. The association between the presence of allele 334 and **relationship satisfaction** became significant, *b* = 0.32, *SE* = 0.14, *t*(85.69) = 2.14, *p* = .035. Notably, White participants reported higher levels of satisfaction than participants from other racial/ethnic groups, *b* = -0.66, *SE* = 0.22, *t*(120.35) = -3.03, *p* = .003, and participants for whom this was the first marriage demonstrated a nonsignificant trend for higher marital satisfaction than participants who have been married previously, *b* = -0.37, *SE* = 0.19, *t*(98.16) = -1.91, *p* = .059. Furthermore, the association between presence of allele 334 was not moderated by racial/ethnic group (*p* = .147) or prior marriage (*p* = .418).

The negative association between the presence of allele 334 and **problems in pair-bonding** remained significant, *b* = -0.51, *SE* = 0.23, *t*(103.33) = -2.18, *p* = .030. Notably, White participants with 1 or more copies of target allele 334 demonstrated a nonsignificant trend for lower problems in pair-bonding than participants from other racial/ethnic groups, *b* = 0.61, *SE* = 0.31, *t*(120.07) = 1.98, *p* = .050. Furthermore, the association between presence of allele 334 and problems in pair-bonding was not moderated by racial/ethnic group (*p* = .954) or prior marriage (*p* = .770).

However, the association between the presence of allele 334 and the **lack of desire for romantic alternatives** became not significant in the analyses controlling for participant race/ethnicity and prior marriage, *b* = 0.23, *SE* = 0.15, *t*(122.94) = 1.58, *p* = .116. Notably, White participants reported lower desire for romantic alternatives than participants from other racial/ethnic groups, *b* = -0.38, *SE* = 0.17, *t*(94.13) = -2.30, *p* = .024, and participants for whom this was the first marriage demonstrated a nonsignificant trend for greater desire for romantic alternatives than participants who have been married previously, *b* = 0.30, *SE* = 0.17, *t*(114.57) = 1.74, *p* = .084. The association between presence of allele 334 was not moderated by racial/ethnic group (*p* = .778) or prior marriage (*p* = .358).

As in the main analyses reported in the manuscript, none of the other associations between the presence of allele 334 and relationship processes were significant in the analyses including the two covariates (all *p*’s ≥ .148).

# References

Fincham, F. D., & Bradbury, T. N. (1992). Assessing attributions in marriage: The relationship attribution measure. *Journal of Personality and Social Psychology*, *62*(3), 457–468.

Hudson, W. W., Harrison, D. F., & Crosscup, P. C. (1981). A short‐form scale to measure sexual discord in dyadic relationships. *Journal of Sex Research*, *17*(2), 157–174.

McNulty, J. K., Olson, M. A., & Joiner Jr, T. E. (2019). Implicit interpersonal evaluations as a risk factor for suicidality: Automatic spousal attitudes predict changes in the probability of suicidal thoughts. (2019). *Journal of Personality and Social Psychology*, *117*(5), 978–997.

Lambert, N. M., Clark, M. S., Durtschi, J., Fincham, F. D., & Graham, S. M. (2010). Benefits of expressing gratitude: Expressing gratitude to a partner changes one’s view of the relationship. *Psychological Science*, *21*(4), 574–580.

Makhanova, A., McNulty, J. K., Eckel, L. A., Nikonova, L., & Maner, J. K. (2018). Sex differences in testosterone reactivity during marital conflict. *Hormones and Behavior*, *105*, 22–27.

Maner, J. K., Gailliot, M. T., Rouby, D. A., & Miller, S. L. (2007). Can’t take my eyes off you: Attentional adhesion to mates and rivals. *Journal of Personality and Social Psychology*, *93*(3), 389–401.

McNulty, J. K. (2011). The dark side of forgiveness: The tendency to forgive predicts continued psychological and physical aggression in marriage. *Personality and Social Psychology Bulletin*, *37*(6), 770–783.

McNulty, J. K., Meltzer, A. L., Makhanova, A., & Maner, J. K. (2018). Attentional and evaluative biases help people maintain relationships by avoiding infidelity. *Journal of Personality and Social Psychology*, *115*(1), 76–95.

Pfeiffer, S. M., & Wong, P. T. (1989). Multidimensional jealousy. *Journal of Social and Personal Relationships*, *6*(2), 181–196.
